# Preparation, structural characterization, and decomposition studies of two new γ-octamolybdates of 4-methylpyridine

**DOI:** 10.1007/s00706-014-1166-0

**Published:** 2014-04-15

**Authors:** Anna Szymańska, Wojciech Nitek, Dorota Rutkowska-Zbik, Wiesław Łasocha

**Affiliations:** 1 Jerzy Haber Institute of Catalysis and Surface Chemistry PAS, Niezapominajek 8, 30-239 Krakow, Poland; 2 Faculty of Chemistry, Jagiellonian University, Ingardena 3, 30-060 Krakow, Poland

**Keywords:** Crystal structure, Transition metals compounds, Polyoxometalates, X-ray structure determination, Thermal decomposition

## Abstract

**Abstract:**

We synthesized two new γ-octamolybdates, and determined their crystal structures from single-crystal X-ray diffraction data. Orange-yellow tetrakis(4-methylpyridinium) bis(4-methylpyridine)-γ-octamolybdate **1** crystallizes in space group P2_1_/c with *a* = 11.586(2) Å, *b* = 15.526(2) Å, *c* = 16.247(2) Å, *β* = 118.753(1)º, *Z* = 2. White tetrakis(4-methylpyridinium) bis(4-methylpyridine)-γ-octamolybdate hydrate **2** crystallizes in space group C2/c with *a* = 27.086(4) Å, *b* = 11.917(2) Å, *c* = 19.332(2) Å, *β* = 124.427(1)º, *Z* = 4. Results of crystal structure determinations are presented and discussed in this paper. Thermal stability and decomposition studies of the obtained two new γ-octamolybdates were performed using TG/DSC and XRPD methods. Both compounds decomposed with the formation of 4-methylpyridinium β-octamolybdate. The two compounds are pseudo-polymorphs, exhibiting both striking similarities as well as significant differences in their structures and properties.

**Graphical Abstract:**

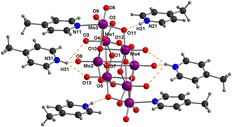

**Electronic supplementary material:**

The online version of this article (doi:10.1007/s00706-014-1166-0) contains supplementary material, which is available to authorized users.

## Introduction

Interest in research connected with polyoxometalates results from the wide range of their potential uses. They are known for their diverse chemical and physical properties, which are important in many areas of science and technology such as medicine, catalysis, electronics, and the production and design of new magnetic materials. These compounds consist of metal–oxygen clusters, where the metal is usually from group 5 or 6 (vanadium, molybdenum, and tungsten are the most common) [1, 2]. Polyoxometalates are used to build new organic–inorganic hybrid materials, which combine characteristic properties of the components with new ones resulting from their synergistic interplay [3]. In view of their catalytic properties, polymolybdates have been studied extensively. Among the polymolybdates, the octamolybdates are the most numerous group. In the Cambridge Structural Database CSD [ver. 5.34], 190 compounds with the octamolybdate anion [Mo_8_O_26_]^4−^, and 335 with the ‘octa-molybdenum’ or ‘octamolybdate’ compound name can be found [4].

**Fig. 1:**
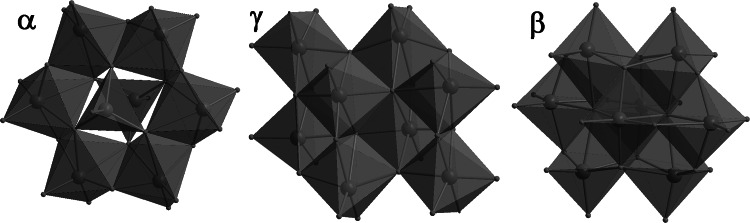
Octamolybdate anions in polyhedral representation

So far, eight isomers of octamolybdates are known: *α*, *β*, *γ*, *δ*, *ε*, *ζ*, *η*, and *θ*. The best-known and also the most commonly occurring is the beta-isomer. Other isomers are much less likely to occur. Construction of the individual isomer is described in the scientific literature [3, 5]. The choice of reaction conditions has a major influence on the formation of a specific form. One such little-known isomer is the γ isomer, which Klemperer and Shum [1] proposed as an intermediate between the α- and β-isomers, to explain the inter-conversion of these forms. It consists of six MO_6_ octahedra and two MO_5_ square pyramid subunits [1, 3]. Moreover, in many cases of Mo atoms, occupying the centrt of MoO_5_ pyramids, organic ligands are coordinated, forming species with the formula Mo_8_O_26_(L_2_)^2n − 4^ (where L denotes ligands other than terminal oxygen, *n* charge of ligand L). Also of interest is an examination of the gamma isomers in terms of their high biological activity [6]. Figure 1 presents three octamolybdate isomers: α, β, and γ.

In our laboratory, two new γ-octamolybdates of 4-methylpyridine have been obtained recently. They contain various amounts of water, thus they are pseudo-polymorphs. To examine the properties of these new compounds, we carried out single-crystal X-ray powder diffraction (to check the purity of the compounds and to study thermal decomposition), thermogravimetry/differential scanning calorimetry (TG/DSC), and infrared (IR) investigations (see “Experimental”).

During thermal decomposition studies, we observed the transformation of γ-octamolybdates into β-isomers. Thus, we decided to investigate the energy and geometry of 4-methylpyridinium γ- and β-octamolybdates by density functional theory (DFT) methods.

## Results and discussion

### Structural studies

In Table 1, the crystallographic data of the two new octamolybates are presented. Both compounds crystallize in a monoclinic crystal system.

**Table 1:** Summary of crystal data of the γ-octamolybdates

Compounds	4-Methylpyridinium octamolybdate(VI) (**1**)	4-Methylpyridinium octamolybdate(VI) hydrate (**2**)
Chemical formula	C_36_H_46_Mo_8_N_6_O_26_	C_36_H_48_Mo_8_N_6_O_27_
Chemical formula (moiety)	[Mo_8_O_26_·2(C_6_H_7_N)]·4(C_6_H_7_NH)	[Mo_8_O_26_·2(C_6_H_7_N)]·4(C_6_H_7_NH)·H_2_O
Formula weight/g mol^−1^	1,746.31	1,764.32
Crystal system	Monoclinic	Monoclinic
Space group	*P* 2_1_/*c* (14)	*C* 2/*c* (15)
Cell parameters/Å, º	*a* = 11.586(2) *b* = 15.526(2) *c* = 16.247(2) *β* = 118.753(1)	*a* = 27.086(4) *b* = 11.917(2) *c* = 19.332(2) *β* = 124.427(1)
Cell volume/Å^3^	2,562.23(6)	5,147.09(13)
*Z*	2	4
Measurement temperature/K	293(2)	100(2)
Calculated density/g cm^−3^	2.264	2.279
*F*(000)	1,696	3,440
Crystal size/mm	0.35 × 0.25 × 0.25	0.35 × 0.30 × 0.15
Crystal color	Orange–yellow	White
Radiation	Mo-Kα	Mo-Kα
*λ*/Å	0.71073	0.71073
Absorption coefficient/mm^−1^	1.983	1.977
2*θ* range	2.90–30.02	2.84–27.48
Collected reflections	24,507	20,482
Unique reflections	7,489	5,891
Observed reflections	6,386	5,427
*R*_1_/*wR*_2_ all data	0.0360/0.0729	0.0252/0.0564
*R*_1_/*wR*_2_ gt *I* > 2*σ*(*I*)	0.0279/0.0683	0.0222/0.0553

The same type of octamolybdate anion is observed in both compounds. The anions are centrosymmetric, as each Mo atom is placed in the center of a distorted MoL′_6_ (L′ = O or N) octahedron. As in most compounds built of MoO_6_ octahedra, in each MoO_6_ octahedron there are two short (~1.75 Å), two medium (~1.95 Å), and two long (~2.25 Å) Mo–O bond distances. Four of the eight Mo atoms possess two terminal oxygen atoms O_T_, while two have only one O_T_ atom. The remaining two Mo atoms (at opposite ends of the anion), besides two terminal oxygen atoms, possess one direct Mo–N bond. To summarize, the anion formula can be given as [Mo_8_O_26_L_2_]^4−^, where in each anion there are 14 terminal O_T_ atoms, 6 *μ*_2_-O, 4 *μ*_3_-O and 2 *μ*_4_-O, and L is 4-methylpyridine. According to the literature (see also Fig. 1), obtained molybdates belong to the group classified as γ-octamolybdates [3].

Porai-Koshits and Atovmyan [7] have claimed that in the polymetalate anion built of Me(VI)O_6_ octahedra, there can be no more than two terminal oxygen atoms, and if such atoms are present, they should be in a *cis* position to each other. In the case of the investigated compounds, even though two MoO_5_ square pyramids are converted to MoO_5_N octahedra, we still have only two terminal oxygen atoms in *cis* position to each other. Thus, the Porai-Koshits and Atovmyan rule is still maintained. A more precise description of the structures of the investigated compounds is presented below.

### Tetrakis(4-methylpyridinium) bis(4-methylpyridine)-γ-octamolybdate (**1**)

**Fig. 2:**
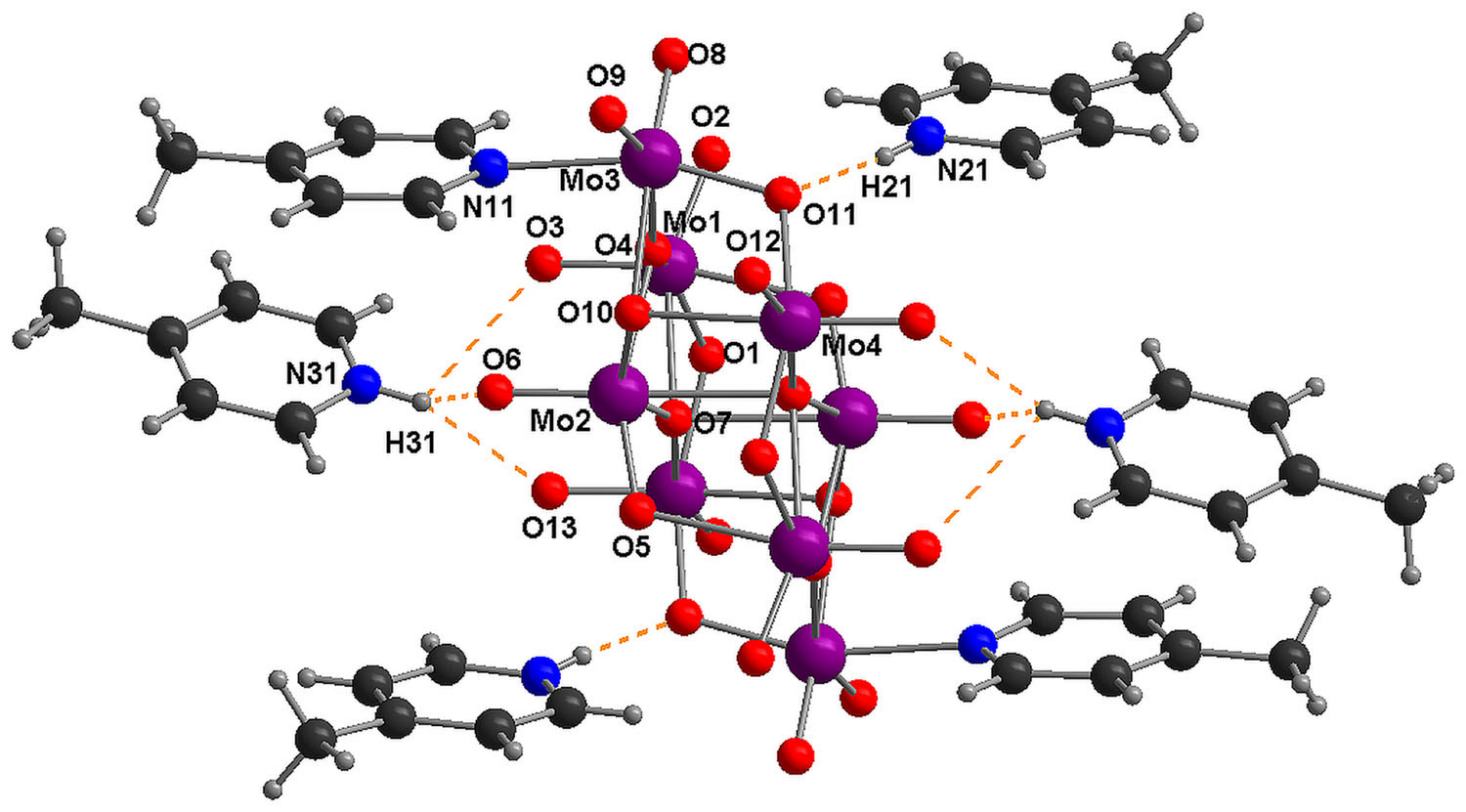
Asymmetric unit and labeling scheme for the symmetry-independent atoms of 4-methylpyridinium octamolybdate(VI) (**1**). The *dashed lines* indicate the hydrogen bonds

**Fig. 3:**
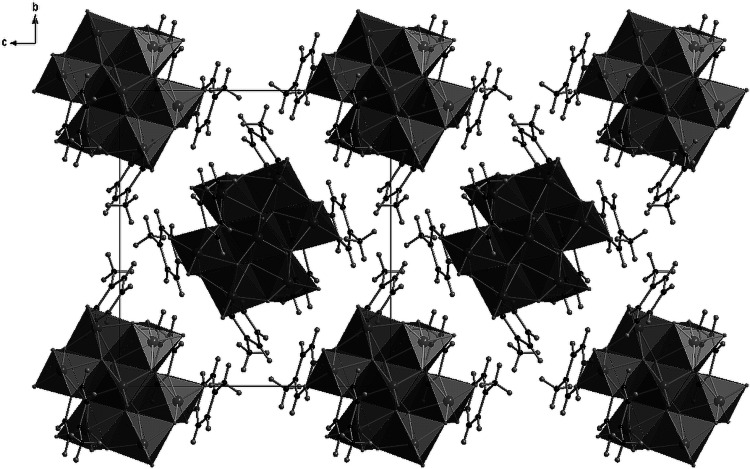
Projection of the structure of 4-methylpyridinium octamolybdate(VI) (**1**) along *b* axis

In the structure of this compound, each centrosymmetric γ-octamolybdate anion is directly connected to two molecules of 4-methylpyridine by a nitrogen–molybdenum bond (N11–Mo3, 2.249(2) Å). Molecules of protonated amines are connected with octamolybdate anions through hydrogen bonds formed by nitrogen from 4-methylpyridine and oxygen atoms from γ-[Mo_8_O_26_]^4−^ anion. In particular, N21 atoms, from the N21..C27 ring, form strong hydrogen bonds (N21…O11, d(D..A) = 2.668 Å), whereas N31 atoms form trifurcated bonds N31…O6, N31…O3, and N31-H…O13 with d(D..A) = 2.89, 3.01, and 3.169 Å, respectively. As can be expected, trifurcated H-bonds are weaker than single bonds created by atoms in a similar environment. Selected bond distances and details of hydrogen bonds are collected in Tables 2 and 3. The asymmetric unit, labeling scheme for the symmetry-independent atoms, and projection of the unit cell are shown in Figs. 2 and 3. It is noteworthy that each octamolybdate anion is surrounded by four 4-methylpyridinium cations (creating seven-element semi-isolated clusters, where the [Mo_8_O_26_]^4−^ fragment is surrounded by six methylpyridine moieties). What is interesting is that there are no H-bonds linking neighboring amines, as they are mutually interacting only by *π*–*π* interactions (shifted face-to-face). In the structure, one can find infinite channels (along [100], passing through 0, ½, 0, 0 and 0, 0, ½), built of 4-methylpyridine moieties placed almost in parallel to each other in a distance of 3.8–4.25 Å. The γ-octamolybdate anions surrounded by cations form columns along the direction [100] (see Fig. 3).

**Table 2:** Selected bond distances of 4-methylpyridinium octamolybdate(VI) (**1**)

Bond distances/Å			
Mo(1)–O(3)	1.7096(18)	Mo(3)–O(8)	1.708(2)
Mo(1)–O(2)	1.7101(18)	Mo(3)–O(9)	1.7096(19)
Mo(1)–O(4)	1.9262(16)	Mo(3)–O(11)	1.8970(18)
Mo(1)–O(1)	1.9267(17)	Mo(3)–O(4)	2.1217(16)
Mo(1)–O(7)#1	2.2708(16)	Mo(3)–O(10)	2.1900(17)
Mo(1)–O(5)	2.3318(17)	Mo(3)–N(11)	2.249(2)
Mo(2)–O(6)	1.7060(17)	Mo(4)–O(12)	1.6954(19)
Mo(2)–O(5)	1.7445(16)	Mo(4)–O(13)	1.713(2)
Mo(2)–O(10)#1	1.8965(16)	Mo(4)–O(1)#1	1.9079(17)
Mo(2)–O(7)	1.9330(16)	Mo(4)–O(11)	2.0110(18)
Mo(2)–O(4)#1	2.1621(16)	Mo(4)–O(7)	2.2497(16)
Mo(2)–O(7)#1	2.3824(16)	Mo(4)–O(10)	2.3076(16)

Symmetry transformations used to generate equivalent atoms: #1 [−*x* + 1, −y, −z]

**Table 3:** Hydrogen bonds of 4-methylpyridinium octamolybdate(VI) (**1**) (with H⋯A < r(A) + 2.000 Å and <DHA >110°)

D-H	d(D-H)	d(H⋯A)/Å	<DHA/Å	d(D⋯A)/°	A [symmetry codes]/Å
N21–H21	0.73	1.945	170.13	2.669	O11
N31–H31	0.853	2.328	123.61	2.889	O6 [−*x* + 1, *y* − 1/2, −*z* + 1/2]
N31–H31	0.853	2.526	132.84	3.169	O13 [−*x* + 1, *y* − 1/2, −*z* + 1/2]
N31–H31	0.853	2.592	111.17	3.007	O3 [*x*, −*y* − 1/2, *z* + 1/2]

### Tetrakis(4-methylpyridinium) bis(4-methylpyridine)-γ-octamolybdate hydrate (**2**)

**Fig. 4:**
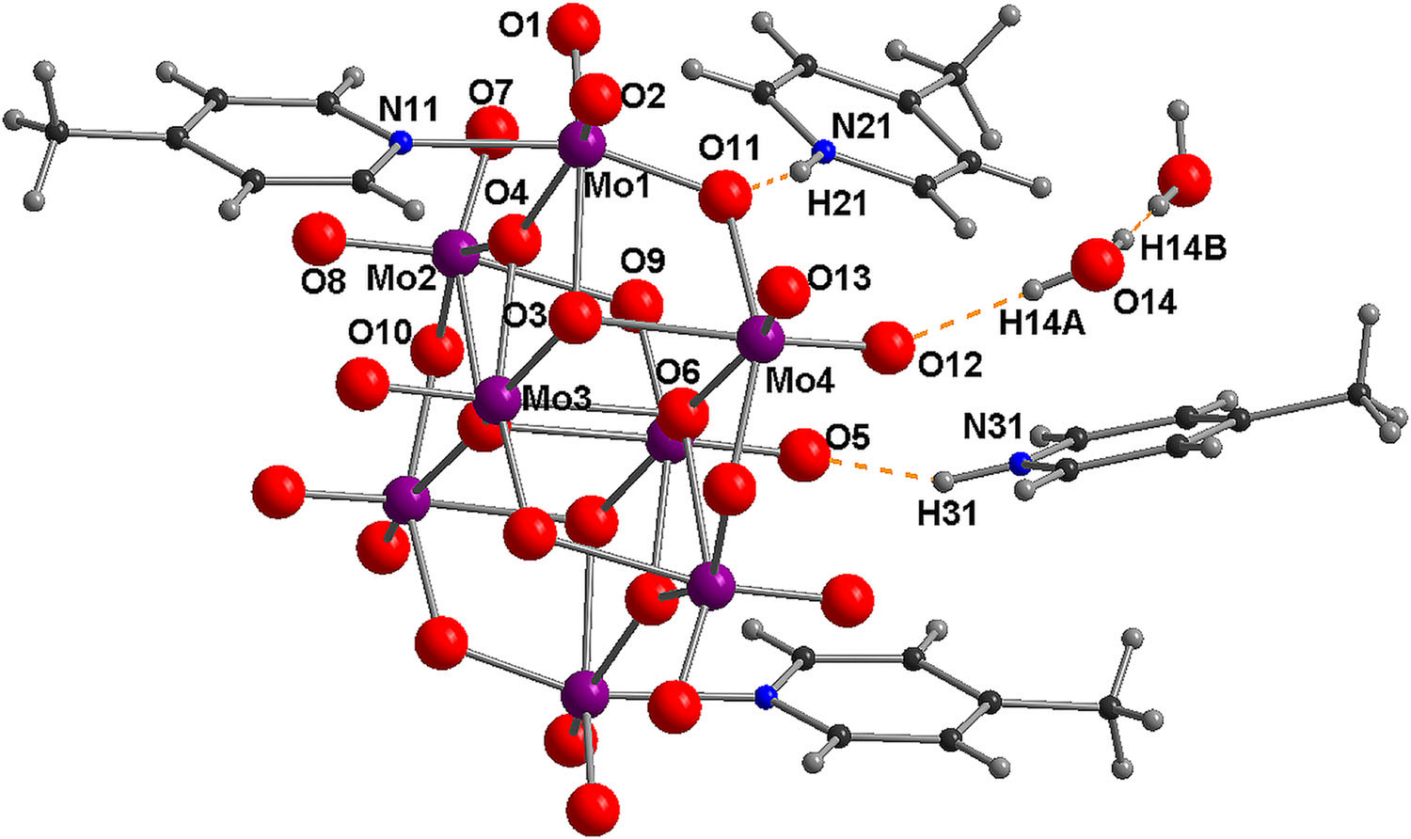
Asymmetric unit and labeling scheme for the symmetry-independent atoms of 4-methylpyridinium octamolybdate(VI) hydrate (**2**). The *dashed lines* indicate the hydrogen bonds

**Fig. 5:**
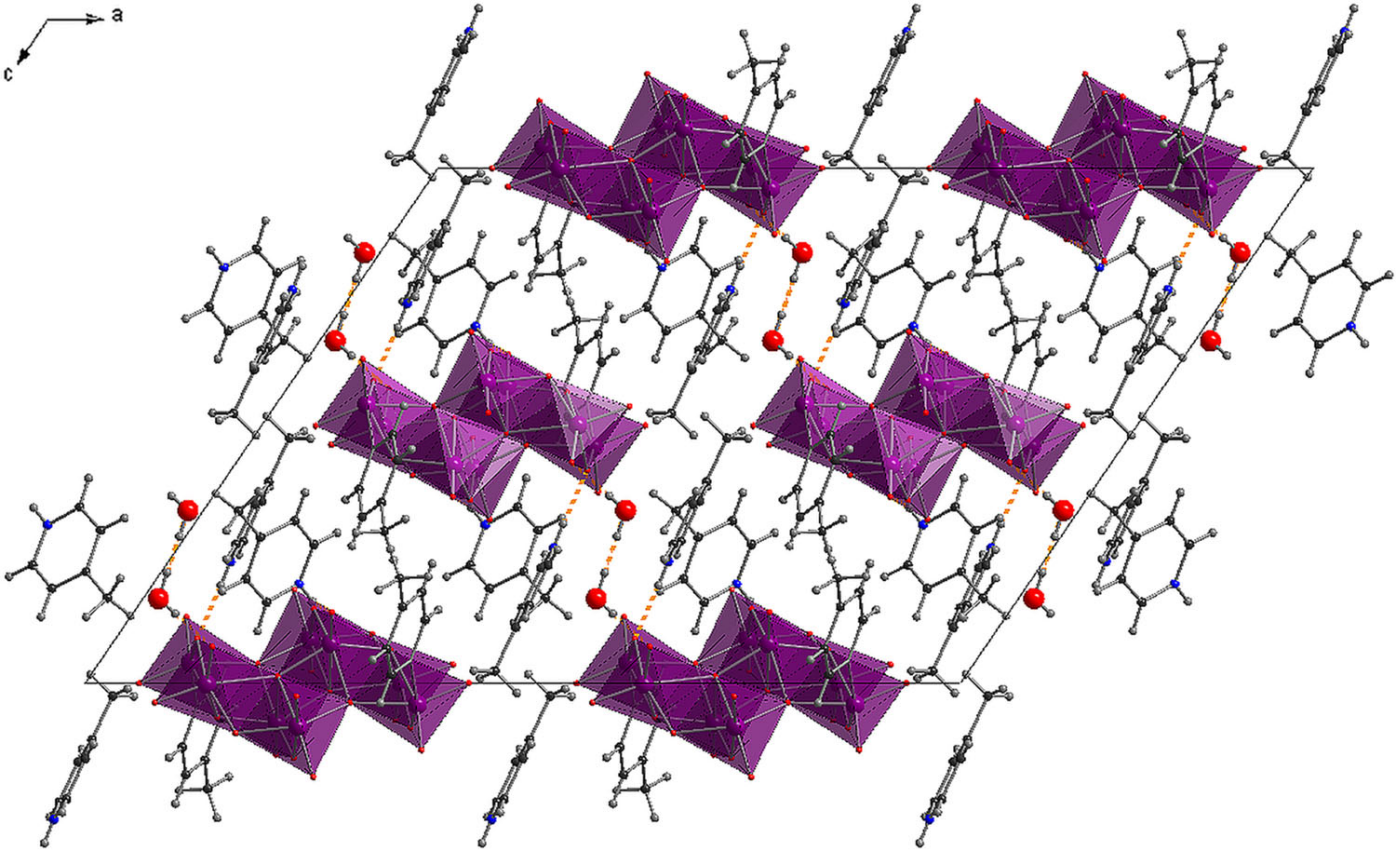
Projection of the unit cell content along the *b* axis of 4-methylpyridinium octamolybdate(VI) hydrate (**2**)

The unit cell of this compound contains four equivalent centrosymmetric γ-octamolybdate anions [Mo_8_O_26_]^4−^, and each one is directly connected to two molecules of amines by a nitrogen–molybdenum bond Mo1–N11 (2.255(2) Å). Figure 4 shows the asymmetric unit and labeling for the symmetry-independent atoms. A projection of the unit cell is presented in Fig. 5. Table 4 contains a list of bond distances. Each anion is surrounded by four 4-methylpyridinium cations. They are connected to the anions by a network of hydrogen bonds, formed by nitrogen atoms from cations and oxygen from octamolybdate anions (N21…O11 2.639 Å, N31…O5 2.685 Å). Furthermore, in the unit cell four water molecules (O14, sof = 0.5) are present, and hydrogen bonds are also formed by oxygen from water molecules and oxygen atoms from [Mo_8_O_26_]^4−^ (O14…O12 2.794 Å, see Table 5). In contrast to **1** (where isolated clusters occur), a system of hydrogen bonds forming infinite layers parallel to (10-1) is observed. Rings (C21…C27) are arranged parallel to the plane (200), creating a double-layer build of 4-methylpyridine molecules.

**Table 4:** Selected bond distances of 4-methylpyridinium octamolybdate(VI) hydrate (**2**)

Bond distances/Å			
Mo(1)–O(1)	1.7064(18)	Mo(3)–O(5)	1.7149(16)
Mo(1)–O(2)	1.7106(18)	Mo(3)–O(9)#1	1.7428(16)
Mo(1)–O(11)#1	1.9018(17)	Mo(3)–O(3)	1.9021(16)
Mo(1)–O(4)	2.1143(16)	Mo(3)–O(6)	1.9345(15)
Mo(1)–O(3)	2.2021(16)	Mo(3)–O(4)	2.1489(16)
Mo(1)–N(11)	2.2554(19)	Mo(3)–O(6)#1	2.3746(15)
Mo(2)–O(7)	1.7071(18)	Mo(4)–O(13)	1.7033(18)
Mo(2)–O(8)	1.7138(17)	Mo(4)–O(12)	1.7165(17)
Mo(2)–O(4)	1.9326(16)	Mo(4)–O(10)	1.9027(18)
Mo(2)–O(10)	1.9385(17)	Mo(4)–O(11)	2.0027(17)
Mo(2)–O(6)	2.2548(16)	Mo(4)–O(3)#1	2.2755(16)
Mo(2)–O(9)	2.3376(16)	Mo(4)–O(6)	2.2936(15)

Symmetry transformations used to generate equivalent atoms: #1 [−*x* + 3/2, −*y* + 1/2, −z + 1]

**Table 5:** Hydrogen bonds of 4-methylpyridinium octamolybdate(VI) hydrate (**2**) (with H⋯A < r(A) + 2.000 Å and <DHA >110°)

D-H	d(D-H)	d(H⋯A)/Å	<DHA/Å	d(D⋯A)/°	A [symmetry codes]/Å
N21–H21	0.793	1.856	169.55	2.640	O11
N31–H31	0.959	1.786	154.68	2.684	O5
O14–H14A	0.849	1.938	179.93	2.787	O12
O14–H14B	0.849	2.027	179.90	2.876	O14 [−*x* + 2, *y*, −*z* + 3/2]

Flattened [Mo_8_O_26_]^4−^ anions are arranged in planes (002) and also in planes parallel to (100), cutting an axis at ¼ and ¾. Organic molecules fill the space between the anions, forming strips parallel to the planes (002), intersecting the z axis at the 0, 0, ¼ and 0, 0, ¾ points (see Fig. 5).

As can be found in Table 1, densities of both compounds are very similar and, as can be expected, are significantly lower than density of 4-methylpyridine β-octamolybdate equal to 2.612 g/cm^3^ [8]. In compound **1**, similarly to anhydrous β-octamolybdate [8], a very simple system of H-bonds is created, leading to discrete units of formula [4-MePyH]_4_[Mo_8_O_26_(4-MePy)_2_]. In hydrated compound **2**, water molecules enable the formation of infinite H-bonds network.

### TG/DSC studies

Results of thermogravimetric studies were interpreted based on weight loss, and thus, only the qualitative, most probable explanations will be given. However, to clarify the processes of thermal decomposition of investigated γ-octamolybdates (also due to scarcity of literature data for this type of compounds), we performed a synthesis of tetrakis(4-methylpirydine) β-octamolybdate [8], identical to that described in the ICDD database (56–1,511). This compound does not contain water or organic groups directly connected to the Mo atoms. In its structure there are similar anions and exactly the same cations (protonated molecules of 4-methylpyridine) as were in investigated γ-octamolybdates. As it turned out, such a compound decomposes in two stages. The first weight loss is observed in the range 250–290 °C, the second one at temperatures around 350–480 °C (Supplementary Material, Fig. 1S). X-ray measurement vs. temperature indicates that until 200 °C, the compound is stable, while the amorphous phase occurs in temperature range 225–250 °C, and molybdenum oxide starts forming at 275 °C (Supplementary Material Fig. 2S). It turns out that the thermal decomposition of γ-octamolybdates proceeds through the β-octamolybdate phase, which is why testing the thermal behavior of β-Mo_8_O_26_^4−^ was also very important.

**Fig. 6:**
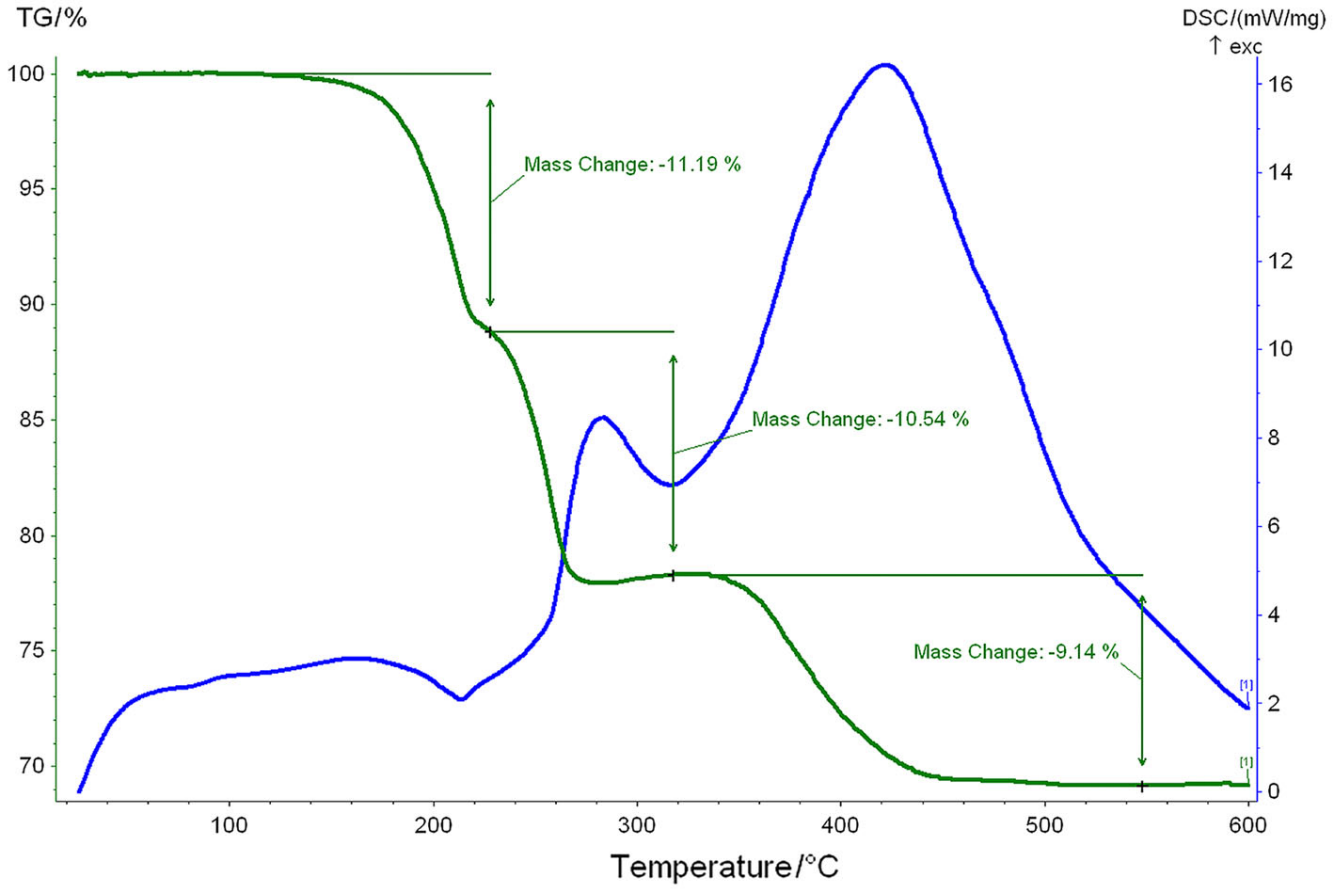
TG/DSC plots of 4-methylpyridinium octamolybdate(VI) (**1**)

The thermal behavior of 4-methylpyridinium γ-octamolybdate(VI) **1** is shown in Fig. 6. The degradation proceeds in three main steps with weight losses of 11.19, 10.54, and 9.14 %. Until the temperature of 150 °C is reached, the compound is stable, and between 150–220 °C, the loss of two amines directly connected to the molybdate anion is observed. The next decomposition stage at ~245 °C is connected with the loss of two protonated molecules of 4-methylpyridinium and an oxygen atom from an octamolybdate anion. The third step of decomposition (350–550 °C) is strongly exothermic, connected with the loss of the other two protonated molecules of amines and the formation of the molybdenum trioxide.

**Fig. 7:**
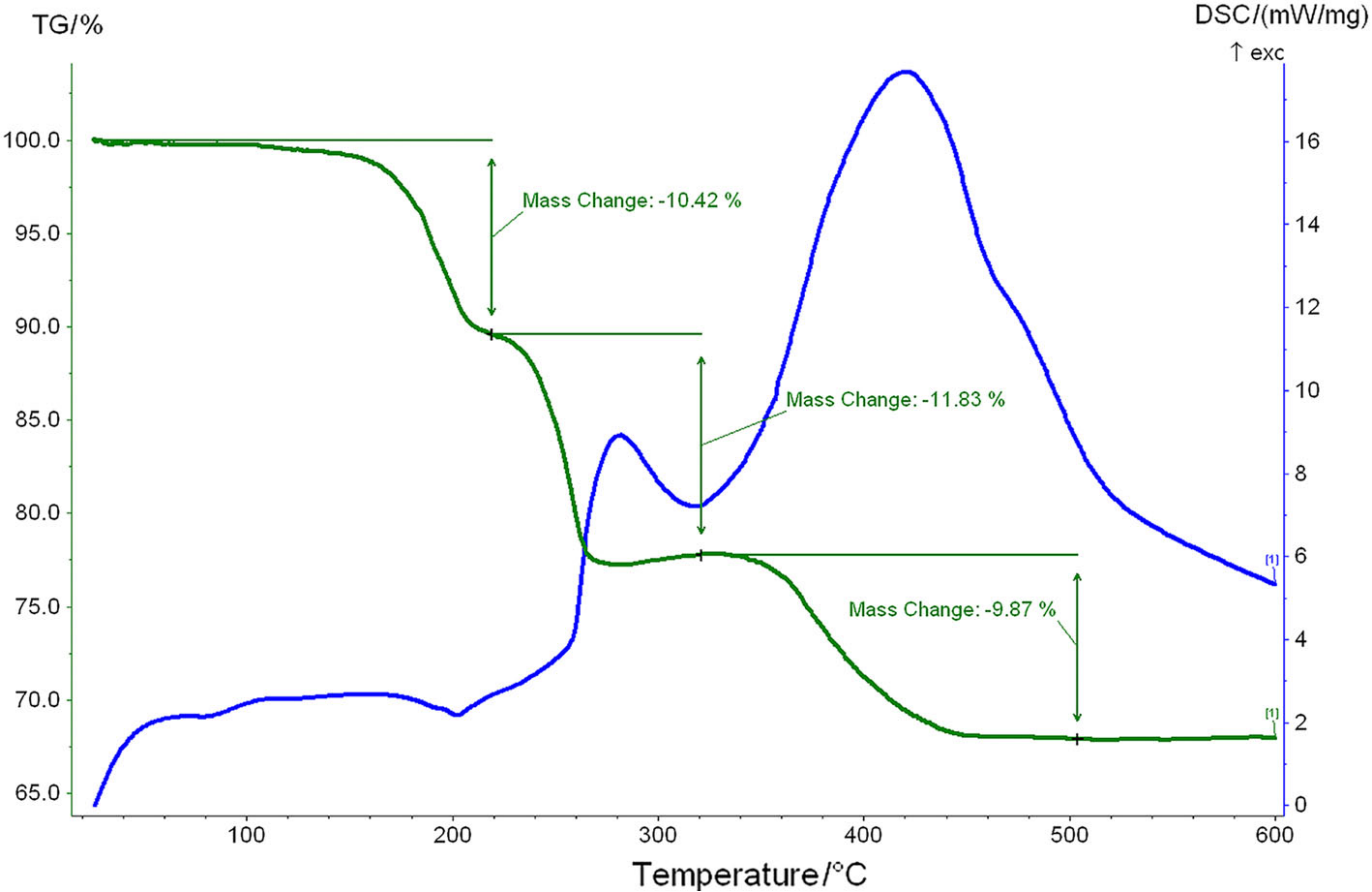
TG/DSC plots of 4-methylpyridinium octamolybdate(VI) hydrate (**2**)

4-Methylpyridinium γ-octamolybdate(VI) hydrate (**2**) decomposes in three main stages (Fig. 7), with weight losses of 10.42, 11.83, and 9.87 %. Nearly equal mass losses at each stage of decomposition correspond to the loss of two 4-methylpyridine molecules per stage.

The loss of water molecules in compound **2** is difficult to notice, however, the total mass change is higher than in compound **1**. Additionally, the first decomposition effect is more diffuse and is observed at a lower temperature than in compound **1**. As can be expected, compounds **1** and **2**, differing only in the content of two water molecules, should exhibit similarities in thermal decomposition, even though they create different structures (with completely different unit-cell parameters and different powder diffraction patterns).

### XRD thermal decomposition studies

The behavior of new γ-octamolybdates in higher temperatures using the X-ray powder diffraction technique was also investigated. In Fig. 3S (Supplementary Material), the thermal decomposition of anhydrous γ-octamolybdate **1** is presented. It turns out that in the temperature range of 150–175 °C, compound **1** transforms into tetrakis(4-methylpyridine) β-octamolybdate ([8], PDF 00-056-1511) and then decomposes at 200 °C into its amorphous phase. Eventually, the crystallization of MoO_3_ above 250 °C is observed.

The decomposition process of 4-methylpyridinium octamolybdate(VI) **2** hydrate goes similarly (Supplementary Material Fig. 4S), except that at 125 °C, both phases of γ- and β-octamolybdates are present, but at higher temperatures only the β isomer occurs. At temperatures of 225–300 °C, the amorphous phase is observed, and in the end MoO_3_ crystallizes.

### IR investigations

Analysis of IR spectra shows that the investigated compounds may differ only in the content of water (characteristic bands coming from vibrations connected with water molecules are present at 3,550–3,200 and 1,630–1,600 cm^−1^ regions [9]). In the case of 4-methylpyridinium γ-octamolybdate hydrate (**2**), bands around 3,500 and 1,680 cm^−1^ are clearly present, while for compound **1** and the beta-isomer, there are almost no bands in this region. A list of IR vibrations for both compounds is given in the Supplementary Material (Table 1S).

### Stability and energy calculations: Results for β- and γ-octamolybdates

The structure and stability of octamolybdate anions [Mo_8_O_26_]^4−^ were tested using the DFT method and the crystal structure parameters of 4-methylpyridinium γ-octamolybdate (**1**) and β-octamolybdate [8].

It was found that the structure of β-octamolybdate [8] is retained after geometry relaxation, regardless of the function. This is confirmed by geometric parameters such as bond lengths and angles. Table 2S summarizes types of coordination polyhedra and the number and type of oxygen atoms, in order to monitor the results of calculations. Calculations indicate that the β-octamolybdate anion is stable and not very susceptible to the influence of external factors.

The XRPD study indicates the transition of γ-octamolybdates into β-octamolybdates at *T* ≥ 150 °C. The calculations confirm that γ-octamolybdate anions (after the loss of two 4-methylpyridine molecules, denoted ‘γ-Mo_8_O_26_^4−^ X-ray’ in Table 6 and 2S) are energetically less favorable than β-anions. Additionally, the optimization of the geometry of the anions (only the inorganic part) through methods of quantum mechanics resulted in different, not yet reported forms of octamolybdate species (see Fig. 6S). Geometry optimizations, therefore, indicate that the transition from γ- to β- form is not a simple, easily realized possibility.

**Table 6:** Energy of β- and γ-octamolybdate anions

Isomer, structure model	Functional, energy/kJ mol^−1^		
	BP	PBE	PW91
β-Mo_8_O_26_^4−^ X-ray	0	0	0
β-Mo_8_O_26_^4−^ optimized	−153.2	−146.5	−42.7
γ-Mo_8_O_26_^4−^ X-ray	126.4	131.5	192.2
γ-Mo_8_O_26_^4−^ optimized	−271.7	−257.9	−44.8
γ-Mo_8_O_26_(L)_2_^4−^ optimized	−126.0	−123.1	−80.4

An attempt was made to verify the geometry of the whole anion containing the 4-methylpyridine moieties (see Table 6 and 2S). It turned out that the organic group stabilizes the anion Mo_8_O_26_(L)_2_^4−^. Table 2S and Fig. 6S show that the optimized γ-octamolybdate anion is essentially the same as the starting model.

In conclusion, in theoretical calculations γ-Mo_8_O_26_(L)_2_^4−^ anions should be treated as a whole. Organic ligands L (here: 4-methylpyridine) may be important stabilizing factors. Their omission from theoretical calculations may determine the results of geometry optimization (Fig. 6S).

In the literature [3], information can be found about the high energy of optimized β-Mo_8_O_26_^4−^ anions compared with gamma forms. Our calculations indicate that the γ-Mo_8_O_26_(L)_2_^4−^ anions we tested are energetically stable only when taking the attached organic components into account. After the loss of organic groups, they become energetically less favorable than the beta form. Evolution of their geometry is difficult to predict, since it is impossible to account in the calculations for all the relevant fragments at a reasonable computational cost.

In addition, it can be stated that in order to optimize the geometry of the investigated Mo_8_O_26_^4−^ anions, the best functional seems to be PW91. By applying it, we get the best models from the viewpoint of crystal chemistry.

Functionals BP and BPE give similar results, taking into account both the energy and geometrical parameters.

## Conclusion

Two new γ-octamolybdates were obtained and characterized, one was synthesized in hydrothermal and one in ambient conditions. In the compounds investigated by us in each centro-symmetric γ-[Mo_8_O_26_]^4−^ anion, two Mo atoms are directly coordinated to N atoms from amines (Mo–N distances 2.24–2.32 Å). The negative charge of the anions is compensated by protonated amines. Gamma octamolybdates can be obtained as anhydrous **1** or hydrated salts **2**.

The direct connection of Mo atoms with amines results in a rather mild temperature at which thermal decomposition begins (150–175 °C). With the release of loosely bonded amines, β-octamolybdate is formed. The final thermal decomposition of the investigated materials occurs around 400–500 °C. In the investigated compounds, this step is connected with a strong exothermic effect.

In the literature, isomer γ is described as a compound that exists in aqueous solution only in inconsiderable amounts [1]. The γ-octamolybdates analyzed and described above were obtained in aqueous solutions in appreciable quantities. We have obtained two methylpyridine octamolybdates, however, the γ-octamolybdate of pyridine (without substituents) is also known [10]. Our syntheses procedures were rather long (more than 24 h) and we used quite large concentrations of reactants (also an excess of 4-methylpyridine in the synthesis of **1**). However, it is still difficult to formulate more general rules, either in favor of or against the synthesis of this kind of compound. It seems that the long reaction time and/or excess of 4-methylpyridine favors the formation of γ-octamolybdates.

Both pseudo-polymorphs **1** and **2** crystallize in different space groups, and their diffraction patterns and crystal structures are different. However, their IR spectra and thermal decomposition processes as observed by TG/DSC are very similar. Both compounds decompose with the formation of a β-octamolybdate phase. In further decomposition steps, amorphous material precedes the formation of MoO_3_, and this phenomenon may be useful in obtaining nanometric MoO_3_ aggregates.

Our DFT calculations indicate that γ-Mo_8_O_26_(L)_2_^4−^ after the loss of the organic group is less stable than the beta form. However, their mutual transformation (γ- into β-octamolybdate) is not a one-step and straightforward process.

## Experimental

### Preparation of γ-octamolybdates

Different types of syntheses, including hydrothermal and crystallization at ambient conditions, were used in our laboratory to obtain new octamolybdates. As a result of our recent efforts, two new γ-octamolybdates were obtained.

#### *4*-*Methylpyridinium octamolybdate(VI)* (**1**, C_36_H_46_Mo_8_N_6_O_26_)

Molybdenum(VI) oxide MoO_3_ (1.44 g, 0.01 mol) and 4.65 g 4-methylpyridine (0.05 mol) were mixed with 15 cm^3^ water. The mixture was heated in a Teflon-lined autoclave for 24 h at a temperature of 150 °C. The solution was then filtered off. Orange–yellow crystals were obtained.

#### *4*-*Methylpyridinium octamolybdate(VI) hydrate* (**2**, C_36_H_48_Mo_8_N_6_O_27_)

Ammonium molybdate tetrahydrate (NH_4_)_6_Mo_7_O_24_·4H_2_O (1.23 g, 1 mmol) was dissolved in 15 cm^3^ water. To the obtained solution 0.2 g 4-methylpyridine (2.15 mmol) was added. The solution was left in a desiccator for crystallization. After a few days, white crystals appeared.

### X-ray data collection

Single crystals were picked up from mother solutions and mounted on the goniometer head. The temperature of the crystals during measurement was 293 K (compound **1**) and 100 K (compound **2**). X-ray data were collected on a *κ*-CCD Bruker–Nonius diffractometer. The multi-scan procedure was performed by diffractometer software for absorption correction. The SHELXS and SHELXL-97 programs [11] were used to solve and refine the structures. All non-hydrogen atoms were refined anisotropically and hydrogen atoms were located from difference Fourier maps. The figures were drawn using the DIAMOND package [12].

CCDC 780058 and 780060 contain the supplementary crystallographic data for **1** and **2**. These data can be obtained free of charge via http://www.ccdc.cam.ac.uk/conts/retrieving.html (or from the Cambridge Crystallographic Data Centre, 12 Union Road, Cambridge CB2 1EZ, UK; fax: +44 1223 336033) email: deposit@ccdc.cam.ac.uk].

### Instrumentation

The stability of the octamolybdates at higher temperatures was investigated using TG/DSC studies. The analyses were carried out on a NETZSCH STA 409 Luxx instrument, at a heating rate of 25 °C/min in an air atmosphere.

Thermal decomposition studies were carried out using a Philips X`Pert Pro MPD powder diffractometer, equipped with a high-temperature chamber Anton Paar. X-ray data were collected at temperatures: 25, 50, 75, 100, 125, 150, 175, 200, 225, 250, 275, 300, 350, 400, and 25 °C again, and the 2*θ* range was from 5° to 65° with a step size of 0.02°.

IR measurements were performed on a Fourier and the vacuum spectrometer Bruker VERTEX 70 V. The samples were pressed into pellets with KBr and investigated at room temperature.

### DFT calculations

In the literature, there are no published data clearly indicating the functionals that should be used in investigations of this type of compounds. It was decided, therefore, to carry out the calculations in parallel, using three functionals: Becke–Perdew (BP) [13–17], Perdew–Becke–Ernzerhof (PBE) [13, 14, 18, 19], and Perdew–Wang (PW91) [18, 20]. The calculations were carried out with the def-TZVP basis set [21], within a resolution of identity approximation (RI) [22, 23], with the use of the Turbomole version 6.2 program [24]. The starting point for the calculations was the crystallographic data. A “single point” type of calculation was performed for the geometry of anions resulting from our structural studies. Then, each of the structures was optimized, allowing all atoms present in the system to fully relax.

## Electronic supplementary material

Below is the link to the electronic supplementary material.

Supplementary material 1 (DOC 6261 kb)
