# Supplementary material for: Preparation, structural characterization, and decomposition studies of two new γ-octamolybdates of 4-methylpyridine
Source: Monatsh Chem. 2014 Apr 15;145(6):921–9. doi: 10.1007/s00706-014-1166-0 (PMC4495051; doi:10.1007/s00706-014-1166-0)
Supplement: Supplementary file 1 — Supplementary material 1 (DOC 6261 kb) [file 706_2014_1166_MOESM1_ESM.doc]

**Supplementary Materials**

**Preparation, structural characterization and decomposition studies of two new γ-octamolybdates of 4-methylpyridine**

**Anna Szymańska^1^ ● Wojciech Nitek^2^ ●Dorota Rutkowska-Żbik^1^ ● Wiesław Łasocha^1,2*^**

^1^ Jerzy Haber Institute of Catalysis and Surface Chemistry PAS, Niezapominajek 8, 30-239 Krakow, Poland

^2^ Faculty of Chemistry Jagiellonian University, Ingardena 3, 30-060 Krakow, Poland

*Corresponding author: nclasoch@cyf-kr.edu.pl

**
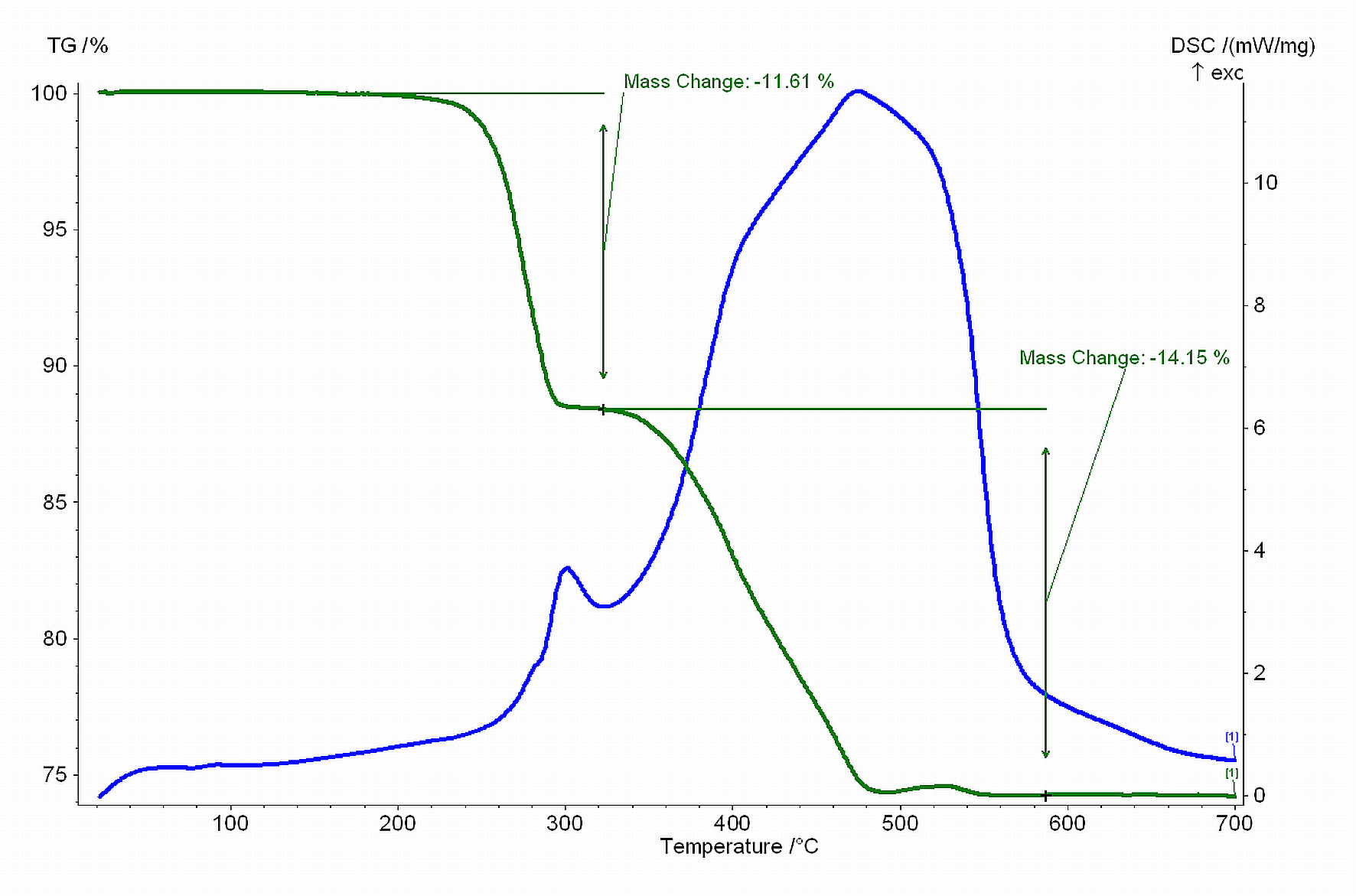
**

Fig. 1S. TG/DSC plots of 4-methylpyridinium β-octamolybdate(VI)


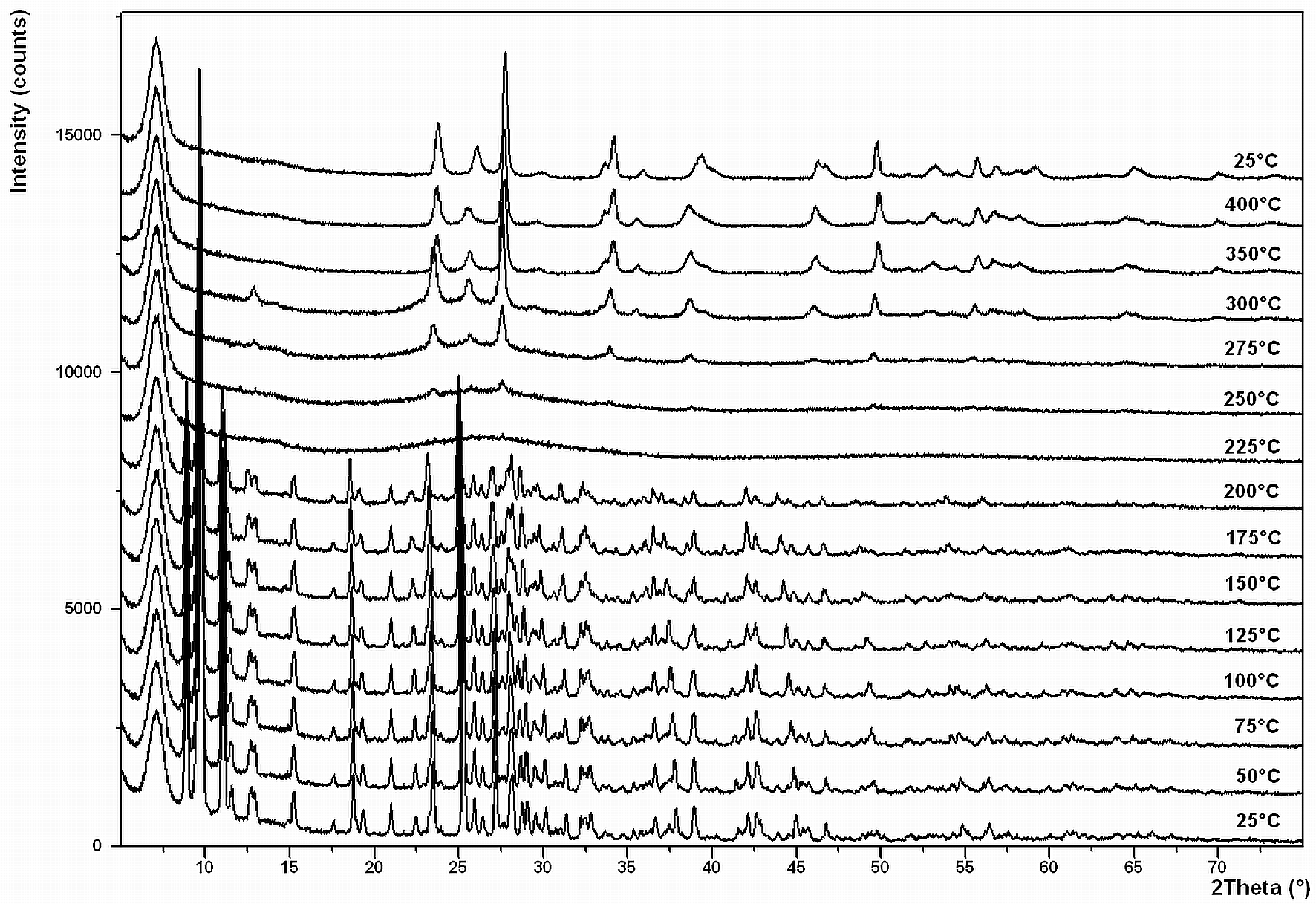


Fig. 2S. XRPD vs. temperature. Thermal decomposition of 4-methylpyridinium β-octamolybdate(VI)

**
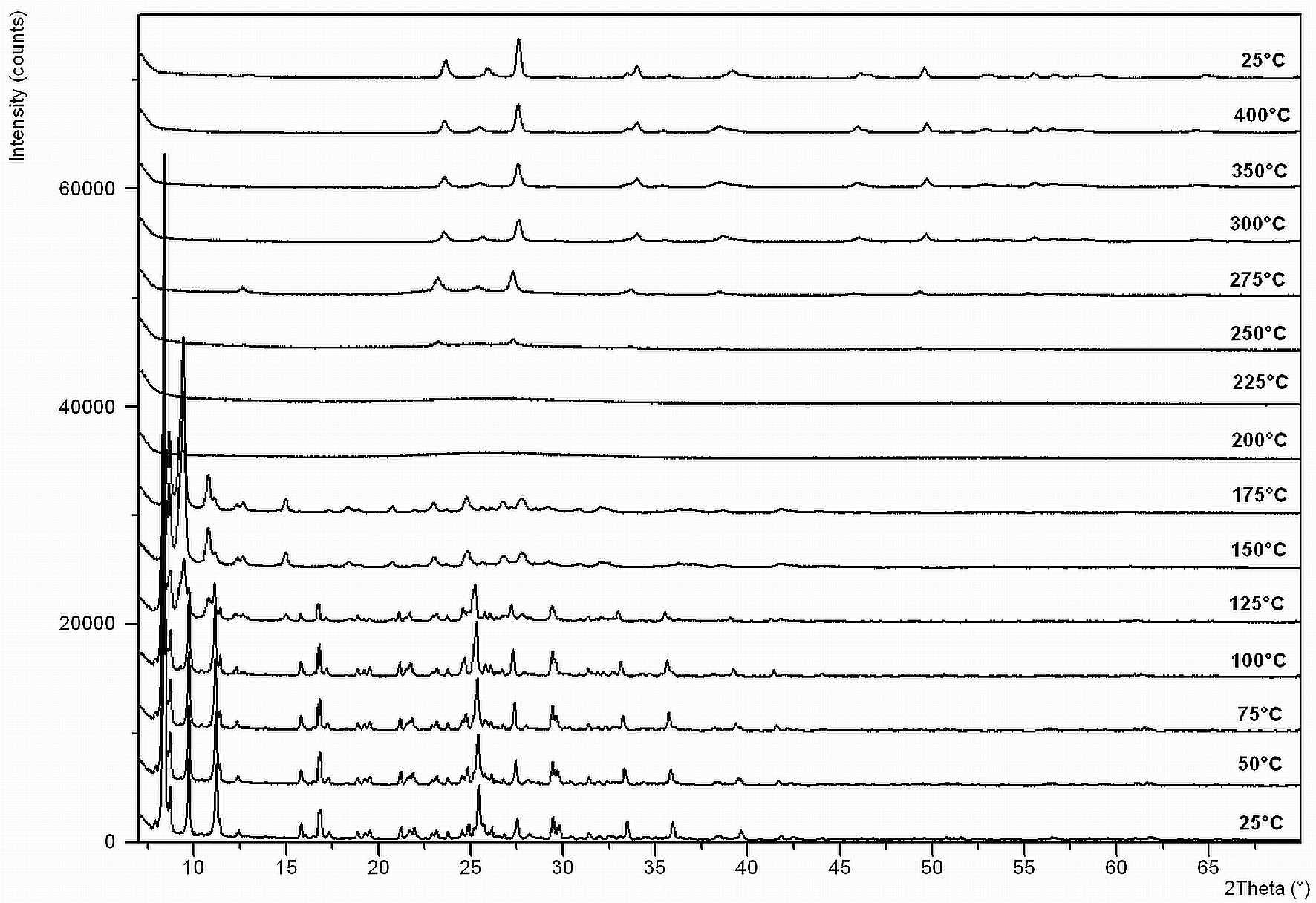
**

Fig. 3S. XRPD vs. temperature. Thermal decomposition of 4-methylpyridinium octamolybdate(VI), compound 1

**
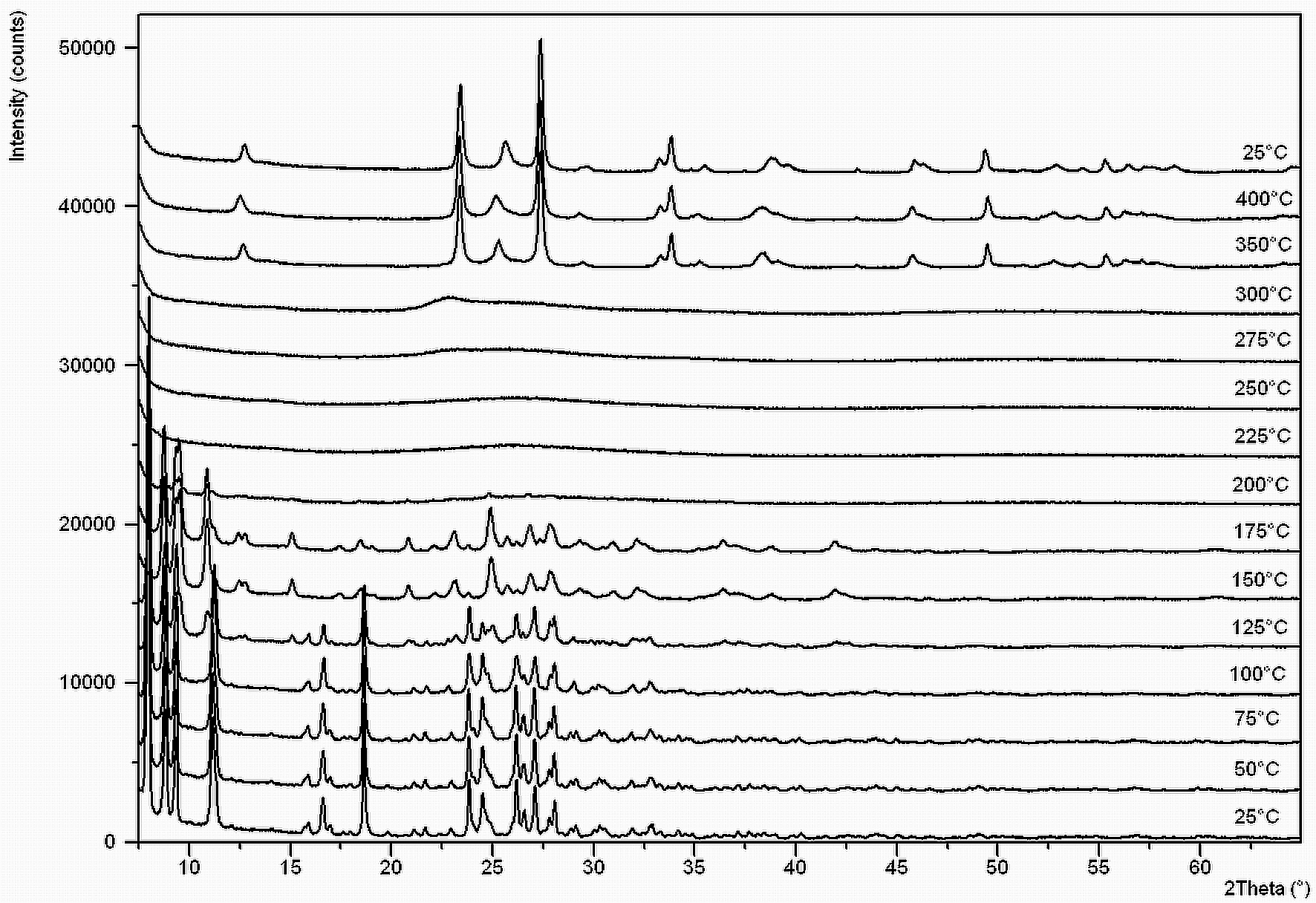
**

Fig. 4S. XRPD vs. temperature. Thermal decomposition of 4-methylpyridinium octamolybdate(VI) hydrate, compound 2


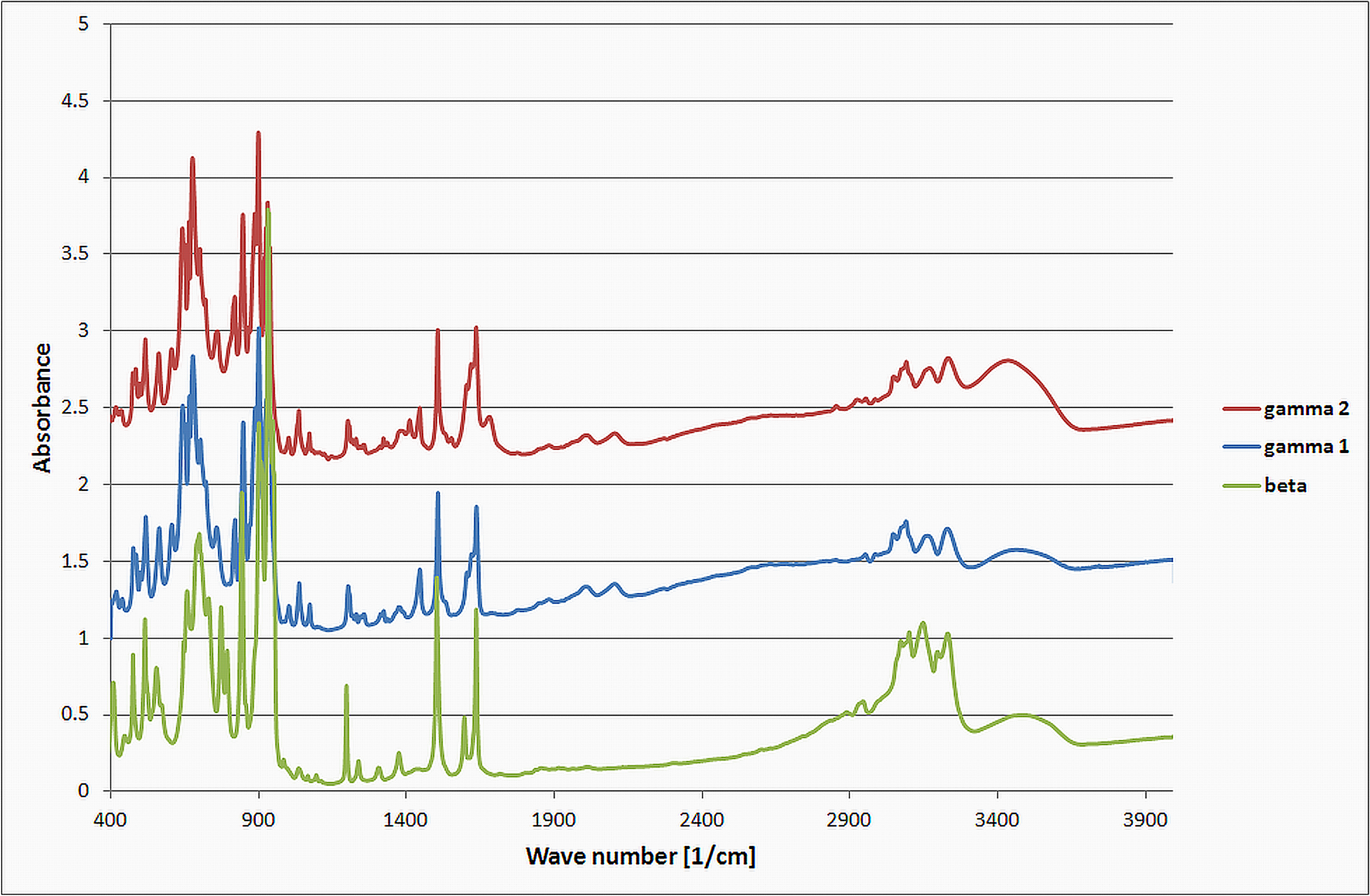


**Fig. 5S.** IR spectra of 4-methylpyridinium octamolybdates, gamma 1 and gamma 2 indicate compound **1** and **2**, respectively. Arrows indicate vibrations caused by hydration water.

**Table 1S. Bands observed in IR spectra of 4-methylpyridinium octamolybdates***

| **Compound** | **Bands** |
| --- | --- |
| 4-methylpyridinium γ-octamolybdate hydrate (2) | 477w; 487w; 499w; 519m; 563m; 607m; 645s; 651s; 665s; 677vs; 704s; 721m; 765m; 820m; 850s; 872w; 887s; 890vs; 930s; 932s; 937s; 1006w; 1037w; 1072w; 1203w; 1233w; 1323w; 1412w; 1446w; 1506m; 1609m; 1627m; 1637m; 1689w; 2021w; 2118w; 3091m; 3179m; 3243m; 3430m |
| 4-methylpyridinium γ-octamolybdate (1) | 473w; 484w; 499w; 518m; 563m; 606m; 643s; 650s; 665s; 676vs; 703s; 717s; 757m; 820m; 847s; 869m; 887s; 900vs; 926s; 930s; 940s; 998w; 1035w; 1069w; 1202w; 1229w; 1248w; 1306w; 1322w; 1374w; 1442w; 1506m; 1604w; 1616w; 1636m; 2008w; 2102w; 3045m; 3089m; 3163m; 3228m |
| 4-methylpyridinium β-octamolybdate | 477w; 517w; 555w; 649w; 662w; 702m; 732w; 775w; 797w; 846s; 903s; 908s; 932vs; 936vs; 949s; 954s; 990w; 1043w; 1067w; 1096w; 1199w; 1238w; 1313w; 1379m; 1504s; 1599w; 1637s; 2952w; 3071m; 3104m; 3153m; 3205m; 3236m |

*w - weak; m - medium; s - strong; vs - very strong

***Stability and energy calculations. Results for β- and γ-octamolybdates***

**Table 2S.** Results of optimization of β- and γ- Mo_8_O_26_^4-^ anions in geometric description

| Isomer and functional | Coordination polyhedra^#^, types and number of oxo-groups | | | | | | | |
| --- | --- | --- | --- | --- | --- | --- | --- | --- |
|  | OCT | TET | SQP | O_T_ | μ2-O | μ3-O | μ4-O | μ5-O |
| γ-octamolybdate: |  | | | | | | | |
| γ-Mo_8_O_26_^4-^ X-ray | 6 |  | 2 | 14 | 6 | 4 | 2 |  |
| γ-Mo_8_O_26_^4-^ BP | 2 | 4 | 2 | 14 | 12 |  |  |  |
| γ-Mo_8_O_26_^4-^ PBE | 2 | 4 | 2 | 14 | 12 |  |  |  |
| γ-Mo_8_O_26_^4-^ PW91* | 6 |  | 2 | 14 | 6 | 4 | 2 |  |
| γ-Mo_8_O_26_(L)_2_^4-^  BP*, PBE*, PW91 | 6 |  | 2 | 14 | 6 | 4 | 2 |  |
| β-octamolybdate: |  | | | | | | | |
| β-Mo_8_O_26_^4-^ X-ray | 8 |  |  | 14 | 6 | 4 |  | 2 |
| β-Mo_8_O_26_^4-^ BP* | 8 |  |  | 14 | 6 |  |  | 2 |
| β-Mo_8_O_26_^4-^ PBE* | 8 |  |  | 14 | 6 |  |  | 2 |
| β-Mo_8_O_26_^4-^ PW91 | 8 |  |  | 14 | 6 | 4 |  | 2 |

^#^ OCT, TET, and SQP denote the number of Mo atoms in octahedral, tetrahedral or square tetragonal pyramid coordination.

* - values obtained assuming Mo-O ≤ 2.75Å as a bonding distance.

γ-Mo_8_O_26_^4-^ PB γ-Mo_8_O_26_^4-^ PBE γ-Mo_8_O_26_^4-^ PW91


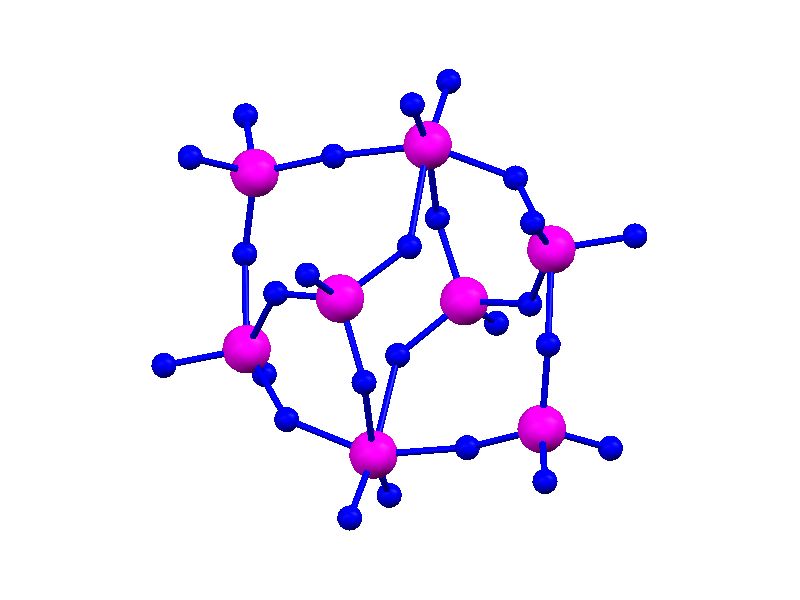

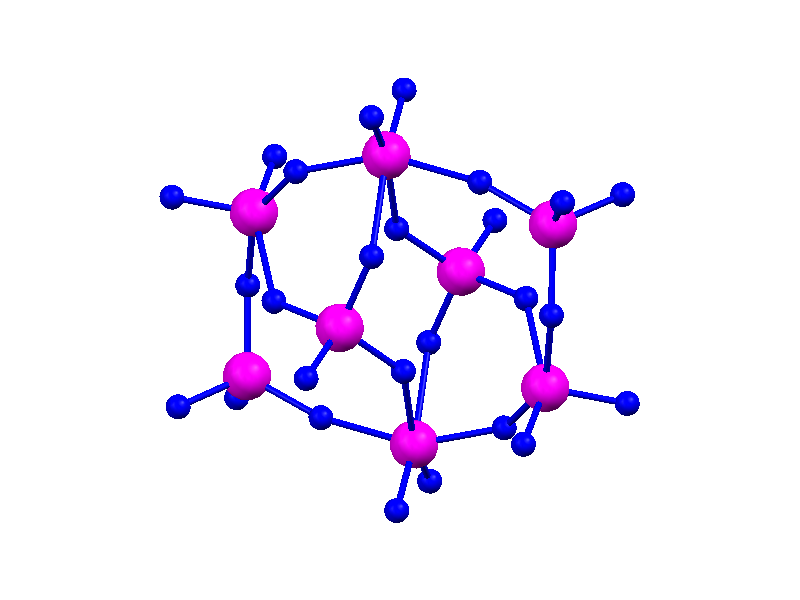

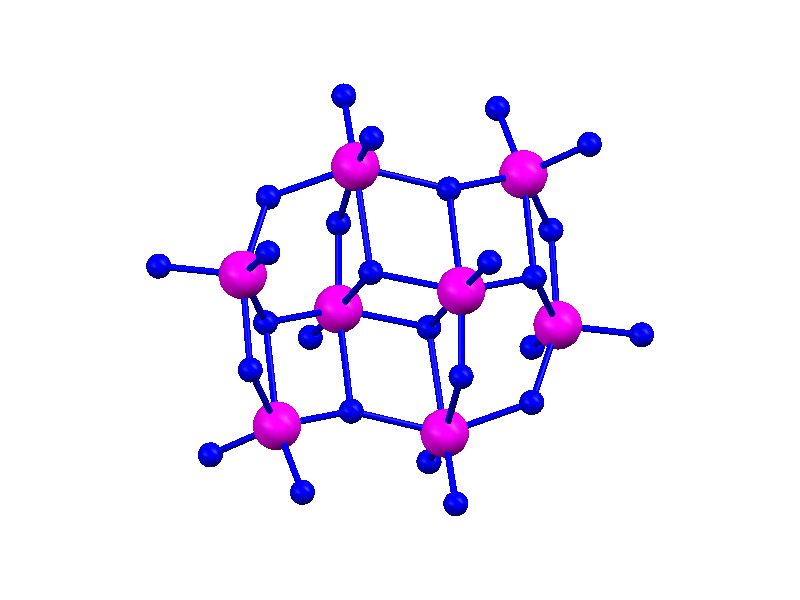


β-Mo_8_O_26_^4-^ PB β-Mo_8_O_26_^4-^ PBE β-Mo_8_O_26_^4-^ PW91


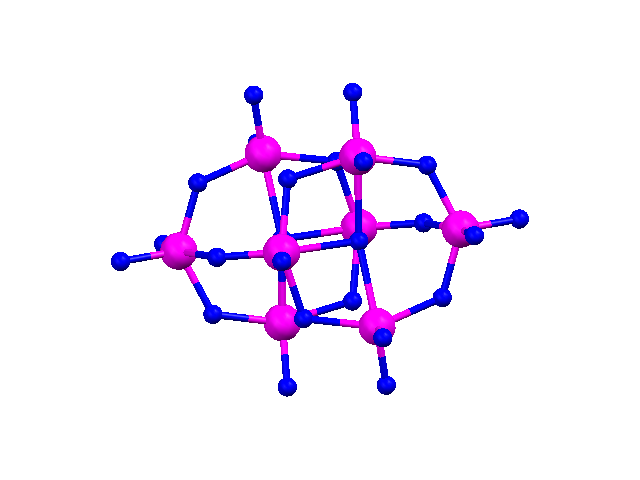

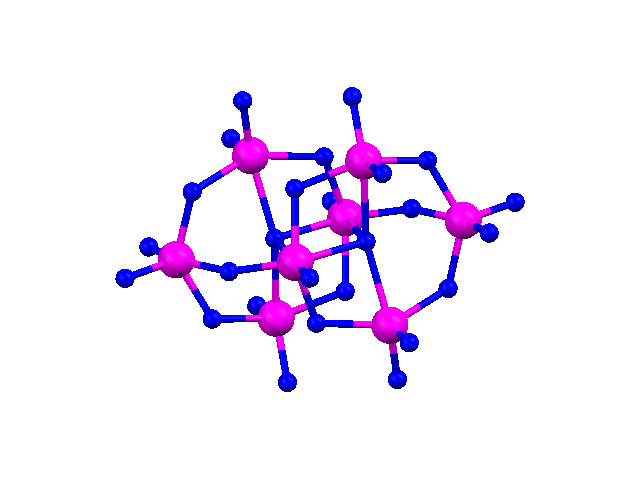

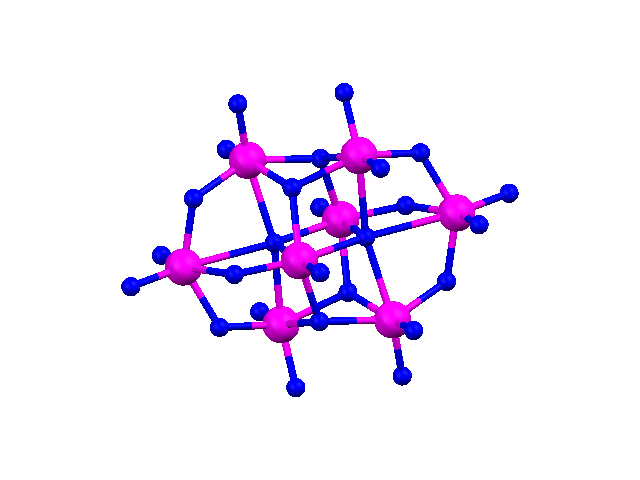


γ-Mo_8_O_26_(L)_2_^4-^ PB γ-Mo_8_O_26_(L)_2_^4-^ PBE γ-Mo_8_O_26_(L)_2_^4-^ PW91


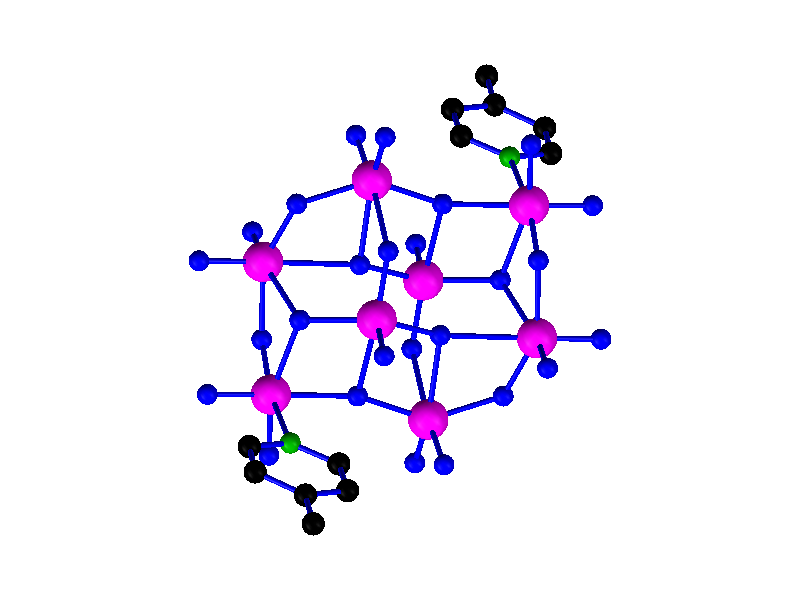

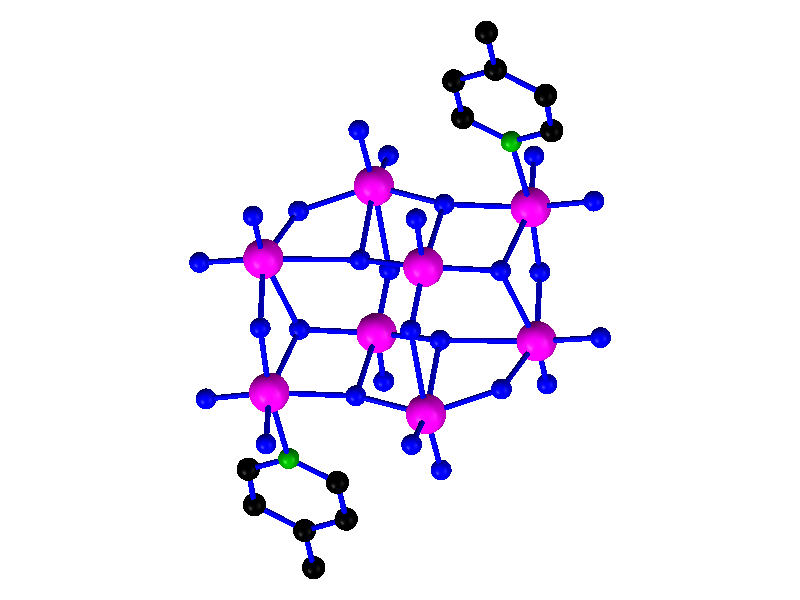

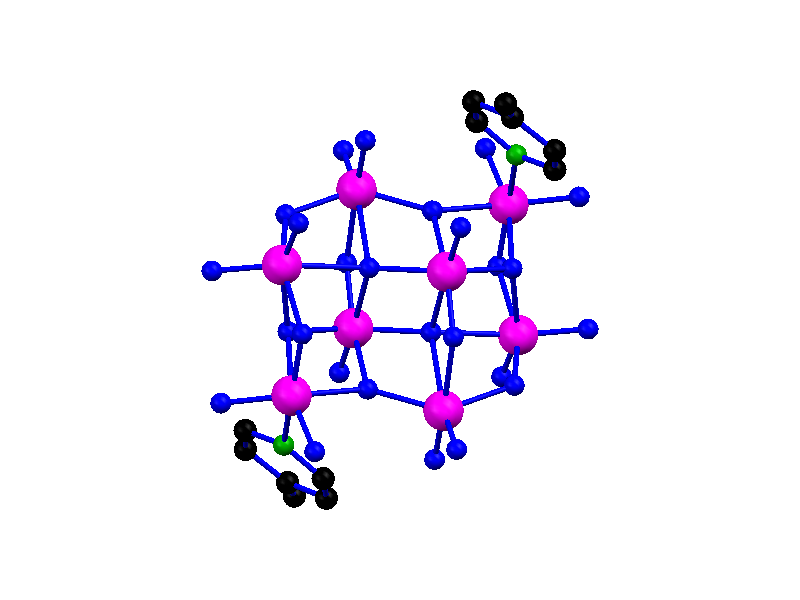


β-Mo_8_O_26_^4-^ X-ray γ-Mo_8_O_26_^4-^X-ray γ-Mo_8_O_26_(L)_2_^4-^ X-ray


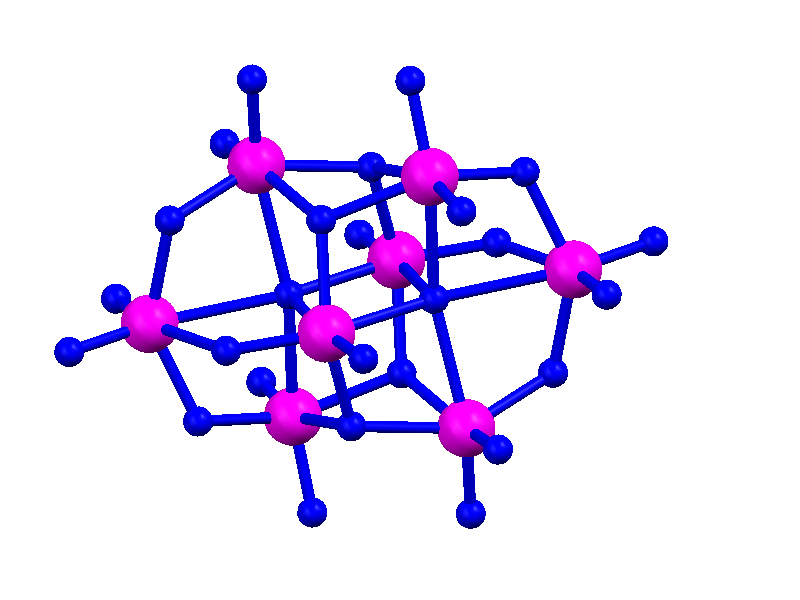

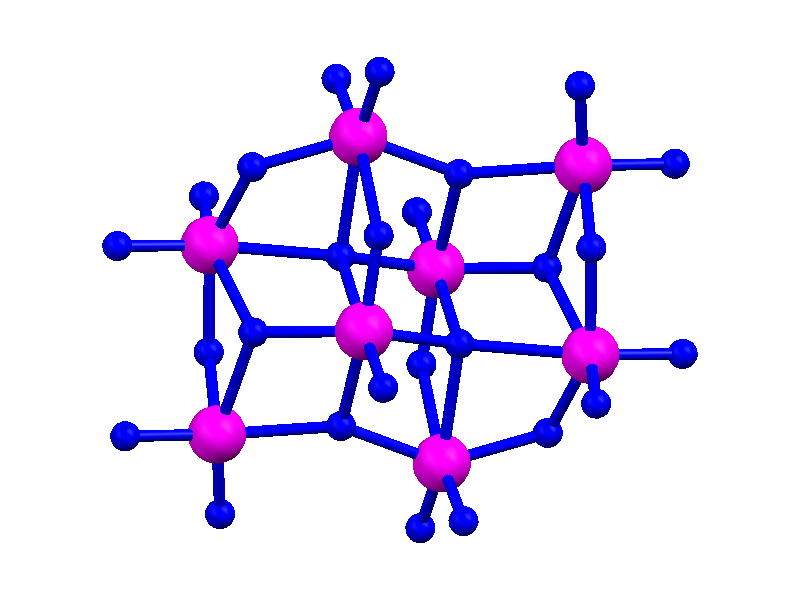

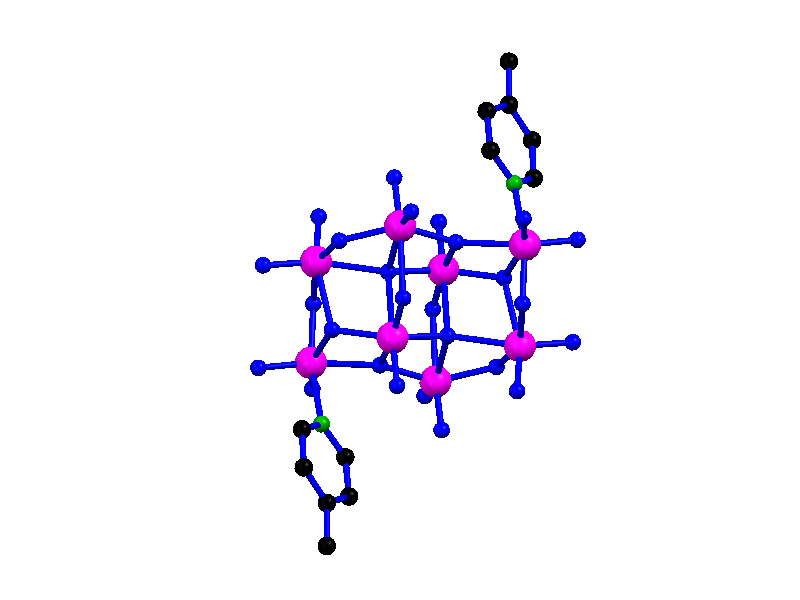


Fig. 5S. Pictures presenting results of geometry optimisation for 4-methylpyridine γ- and β-octamolybdates. Labels are the same as in Tables 6 and 2S.
